# Outcomes of spontaneous pneumothorax in neonates: treatments vs. expectant management

**DOI:** 10.1038/s41372-025-02259-4

**Published:** 2025-03-15

**Authors:** Anat Margaliot, Laurence Mangel, Yarden Waxman, Moria Be’er, Ronella Marom, Jacky Herzlich

**Affiliations:** 1 Department of Neonatology, Tel Aviv University, Tel Aviv, Israel; 2 Pediatric Pulmonology Unit, Tel Aviv University, Tel Aviv, Israel; 3 Dana Dwek Children’s Hospital, Tel Aviv Sourasky Medical Center, Faculty of Medical & Health Sciences, Tel Aviv University, Tel Aviv, Israel

**Keywords:** Respiratory tract diseases, Respiration

## Abstract

**Background:**

Neonatal pneumothorax is dependent on severity of symptoms and leak size. Treatment of Pneumothorax with Nitrogen washout is controversial.

**Objective:**

To compare outcomes of neonates treated for spontaneous pneumothorax (SP) compared with those managed expectantly.

**Methods:**

A retrospective review of medical records of neonates (≥34weeks) diagnosed with SP born between 2011 and 2021. Neonatal characteristics and outcomes were compared between neonates treated for SP with either targeted oxygen therapy (to stabilize saturation ≥93%) or nitrogen washout and those under expectant management.

**Results:**

Among neonates with respiratory distress and desaturation (*n* = 64), nitrogen washout reduced median time to resolution compared to targeted oxygen therapy (31 [12–67] vs 81 [31.8–123.5] hours, *p* = 0.012). Expectantly managed neonates with distress (*n* = 87) experienced delayed feeding initiation, while time to resolution and hospital stay were similar.

**Conclusion:**

Nitrogen washout is superior to targeted oxygen therapy in time to resolution in infants presenting with respiratory distress and desaturation.

## Introduction

Air leakage, which is more frequently seen in newborns than at any other life stage, occurs when over-distended alveoli rupture into the perivascular bundle, allowing air to reach the pleura causing a pneumothorax [1–3]. The prevalence of radiologic spontaneous pneumothorax (SP) is 1 to 2% and symptomatic SP, is 0.05 to 1% in all live births, typically occurring within the first hours of life [4–6].

The SP can be a primary event, occurring in patients with no underlying lung pathology or a secondary event which is a complication of preexisting lung conditions such as meconium aspiration syndrome (MAS), pneumonia, respiratory distress syndrome (RDS), diaphragmatic hernia, or pulmonary hypoplasia [7, 8]. Management of neonatal pneumothorax is dependent on severity of symptoms, air leak size, and is at the discretion of the attending neonatologist at the time of event. There are currently no specific guidelines regarding the treatment of pneumothorax in newborns.

Research on adults has indicated that inhaling elevated levels of oxygen (ranging from 60% to 100%) boosts the rate at which symptomatic SP resolves compared to breathing normal air [9–11]. The underlying principle of oxygen therapy lies in its ability to lower the partial pressure of nitrogen in the alveoli near the pleural cavity, thus establishing a diffusion gradient for nitrogen that expedites resolution [12]. Despite limited evidence on the benefits and safety of nitrogen washout treatment of pneumothorax in newborns, it has been adopted as regular practice in many neonatal intensive care units (NICU).

However, there is a growing body of literature highlighting the potential adverse effects of high oxygen treatment in newborns, through the development of reactive oxygen species, altering genetic cell expression, and enhancing cellular apoptosis [13–19].

The aim of this study was to describe the clinical profiles and outcomes of neonates treated for pneumothorax compared to those managed expectantly. Recognizing that pneumothorax can range from mild to life-threatening, we categorized the neonates based on their clinical presentation, which likely reflected the severity of their condition. Our focus was on the common, straightforward cases of pneumothorax encountered in daily NICU practice that did not require needle aspiration, chest tube insertion, or intubation and were defined as mild to moderate pneumothorax. We hypothesized that the time to resolution of pneumothorax would be shorter in neonates treated with nitrogen washout.

## Material and methods

### Participants

We retrospectively reviewed medical records of neonates (≥34 weeks of gestation) diagnosed with pneumothorax, born between 01 January 2011 and 31 December 2021. We chose to study this cohort of preterm infants (GA ≥ 34 weeks) and newborns because they are the most commonly encountered birth group [20, 21]. Our tertiary center has approximately 12,000 live birth per year. The local institutional review board approved this study (0463-22-TLV) and waived the need for informed consent due to its retrospective character. The study was carried out in accordance with Good Clinical Practice guidelines and the Declaration of Helsinki.

### Data collection

Demographic and clinical data of the neonates included gestational age (GA), gender, birth weight (BW), mode of delivery, morbidities (Disseminated intravascular coagulation (DIC), hypo/hyper-glycemia, respiratory distress syndrome (RDS), meconium aspiration syndrome (MAS), small for gestational age (SGA), appropriate for GA (AGA) and large for GA (LGA), [22], medications, respiratory status and treatment, feeding status, X-ray imaging, admission to NICU and length of stay. Maternal demographic and clinical data included morbidities and medications. Time to resolution was defined upon X-ray imaging conducted either the morning following SP diagnosis or immediately after the completion of Nitrogen washout treatment. Neonates were classified by pneumothorax treatments (Targeted oxygen therapy (oxygen supplementation adjusted to saturation to stabilize saturation ≥93%) or Nitrogen washout therapy for 8 hours (90–100% inspired O_2_ concentration) or expectant management (observation only) and further sub-stratified by clinical presentation at time of pneumothorax diagnosis (respiratory distress alone or desaturation in addition to respiration distress). Desaturation was defined as oxygen saturation ≤92%. In accordance with local protocol, oxygen treatment is provided solely in the NICU, where continuous monitoring of oxygen levels is conducted.

### Statistical analysis

Categorical variables are reported as frequencies and percentages and continuous variables as means and standard deviations or median and interquartile range. Normality was assessed by Shapiro-Wilk tests. Mann-Whitney U test or Kruskal-Wallis test was applied to compare continuous variables between the groups when appropriate. Chi-square tests or Fisher’s exact tests were applied to compare categorical variables. Spearman’s rank correlation was applied to assess the strength and direction of association between continuous variables. Linear regressions (Enter or Stepwise) were used to investigate the variability in length of hospital stay and in time to resolution while controlling for possible confounders such as GA, BW, gender, age at diagnostic, RDS, and MAS. IBM SPSS Statistics for Windows, version 29, was used for statistical data analyses and *p* values < 0.05 were considered statistically significant.

## Results

**Fig. 1:**
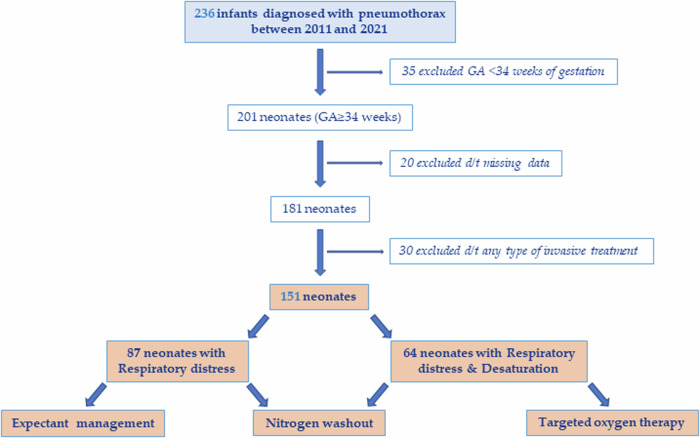
Cohort study.

Of all infants admitted to the NICU and nursery during the 10-year study period, 236 neonates with pneumothorax were eligible. We excluded neonates born before 34 weeks of gestation, those with missing data, those who underwent invasive treatment such as intubation at the delivery room and those who were treated with chest tube or needle after pneumothorax was confirmed by X-ray. The study cohort included 151 cases of mild to moderate pneumothorax, classified based on clinical presentation at time of diagnosis, into two groups: those with respiratory distress (*n* = 87) and those with both respiratory distress and desaturation (*n* = 64) (Fig. 1).

The cohort characteristics are shown in Table 1. Neonates had an average GA of 39 ± 1.7 weeks and an average BW of 3271 ± 509 grams and a third of the cohort were female neonates. Pneumothorax was predominantly diagnosed on the first day of life. Most of the cases (69.5%) were diagnosed as primary event and 72.8% of the neonates were admitted to NICU.

**Table 1:** Characteristics of the cohort (*N* = 151).

Maternal betamethasone treatment	2 (1.3)
Gestational diabetes mellitus	14 (9.3)
Maternal medication	15 (9.9)
Gestational age, w,	38.8±1.7 (34–42)
Birth weight, g,	3271.2±508.8 (1665–4445)
Mode of delivery	
Vaginal	69 (45.7)
Cesarean	66 (43.7)
Vacuum	16 (10.6)
Sex (Female)	45 (29.8)
Twins	4 (2.6)
SGA	8 (5.3)
AGA	125 (82.8)
LGA	18 (11.9)
Age at diagnosis, d,	1 [1-1]
Primary event	105 (69.5)
Secondary event	46 (30.5)
NICU admission	110 (72.8)
Pneumothorax treatment	
Expectant management	53 (35.1)
Targeted oxygen therapy	12 (7.9)
High-concentration oxygen therapy (Nitrogen washout)	86 (57)
Length of stay, d,	4 [3-6]

Data are expressed by median [IQR], mean ± SD (range) or *n* (%)

*AGA* appropriate gestational age, *SGA* small gestational age, *LGA* large gestational age, *NICU* neonatal intensive care unit.

Around forty four percent of the neonates were delivered by cesarean section. High-concentration oxygen therapy (Nitrogen washout) was the predominant treatment performed in 57% of the neonates followed by expectant management (35.1%) and targeted oxygen therapy (7.9%).

Table 2 displays the comparison of demographics and neonatal outcomes across the different treatments (expectant management, nitrogen washout and targeted oxygen therapy). There were no differences between the sub-groups in terms of maternal betamethasone treatment, gestational diabetes mellitus and maternal medication. Additionally, GA, age at diagnosis, gender ratio, mode of delivery, DIC, and hypo/hyper-glycemia were similar. Most of the pneumothorax cases in the expectant management and nitrogen washout sub-groups were primary events (83% and 68.6%, respectively) whereas 83.4% were secondary events in the targeted oxygen therapy sub-group. Most infants, irrespective of their treatment sub-group, did not require ventilation support either prior to or following the pneumothorax event. All of the pneumothorax cases in the expectant management sub-group were characterized by respiratory distress alone whereas desaturation along with respiratory distress characterized 100% and 60.5% of the pneumothorax cases within the targeted oxygen therapy and nitrogen washout sub-groups, respectively. In linear regression analysis, length of hospitalization stay was correlated with age at diagnosis (*B* = 2.05, *t* = 7.45, 95%CI [1.5–2.6], *p* ≤ 0.001), the presence of respiratory distress and desaturation (*B* = 2.21, *t* = 2.8, 95%CI [0.7–3.8], *p* = 0.006], RDS (*B* = 4.16, *t* = 2.89, 95%CI [1.3–7], *p* = 0.004), and maternal betamethasone treatment (*B* = 7.62, *t* = 2.71, 95%CI [2.1–13.2], *p* = 0.008) adjusted for gender, GA, BW, NICU admission, mode of delivery, maternal medications, and SP treatment. The model accounted for a significant proportion of variance in length of hospital stay (R^2^ = 0.484, F(11, 150) = 11.83, *p* < 0.001).

**Table 2:** Characteristics of the neonates by pneumothorax management.

	Expectant management *n* = 53	Targeted oxygen therapy *n* = 12	Nitrogen washout *n* = 86	*p* value
Maternal betamethasone treatment	1 (1.9)	0	1 (1.2)	>0.99
Gestational diabetes mellitus	4 (7.5)	1 (8.3)	9 (10.5)	0.907
Maternal medication	3 (5.7)	3 (25)	9 (10.5)	0.123
Gender (Female)	13 (24.5)	6 (50)	26 (30.2)	0.225
Gestational age, w	39 (38–40)	39 (35.3–40)	39 (38–40)	0.771
Twins	2 (3.8)	1 (8.3)	2 (1.2)	0.187
Mode of delivery				
Vaginal	28 (52.8)	2 (16.7)	39 (45.3)	0.153
Cesarean	21 (39.6)	9 (75)	36 (41.9)	
Vacuum	4 (7.5)	1 (8.3)	11 (12.8)	
SGA	3 (5.7)	1 (8.3)	4 (4.7)	0.791
AGA	42 (79.2)	100 (83.3)	73 (84.9)	
LGA	8 (15.1)	1 (8.3)	9 (10.5)	
Respiratory support at the delivery room				
None	50 (94.3)	10 (83.3)	75 (87.2)	0.299
Ambu resuscitator	3 (5.7)	1 (8.3)	8 (9.3)	
Meconium aspiration syndrome (MAS)	0	1 (8.3)	3 (3.5)	
Admitted to NICU	12 (22.6)	12 (100)	86 (100)	**<0.001**
Age at diagnosis, d	1 (1–1)	1 (1–1)	1 (1–1)	0.112
Respiratory distress alone	53 (100)	0	34 (39.5)	**<0.001**
Desaturation and Respiratory distress	0	12 (100)	52 (60.5)	**<0.001**
Primary event	44 (83)	2 (16.7)	59 (68.6)	**<0.001**
Secondary event				
RDS	1 (1.9)	5 (41.7)	5 (5.8)	< **0.001**
MAS	8 (15.1)	5 (41.7)	22 (25.6)	0.109
Ventilation support				
None	53 (100)	11 (91.7)	83 (96.5)	0.118
Before Pneumothorax	0	0	0	
After Pneumothorax	0	1 (8.3)	3 (3.5)	
DIC	0	0	1 (1.2)	>0.99
Hypoglycemia	1 (1.9)	2 (16.7)	4 (4.7)	0.121
Hyperglycemia	0	0	0	
Length of oxygen therapy, h	0	23.5 (8.5–54.8)	10 (9–16.5)	0.103^a^
Time to resolution, h	36 (23–64)	81 (31.8–123.8)	33 (13.8–63.3)	**0.019**
Length of hospitalization, d	4 (3–5)	6 (4.5–15)	4 (3–7)	**0.001**

Data are expressed by median (IQR), or *n* (%).

*AGA* appropriate gestational age, *SGA* small gestational age, *LGA* large gestational age, *NICU* Neonatal Intensive Care Unit, *RDS* Respiratory Distress Syndrome, *DIC* Disseminated Intravascular Coagulation.

^a^Mann–Whitney test between targeted oxygen therapy and nitrogen washout sub-groups.

Table 3 specifically examines neonates diagnosed with pneumothorax who presented with respiratory distress only, comparing those managed expectantly with those undergoing nitrogen washout. There were no significant differences between these sub-groups in terms of GA, BW, gender ratio, age at diagnosis, and length of hospital stay. However, in the nitrogen washout sub-group, neonates experienced a statistically significant median delay to first feeding of 13.5 hours and a median length of exposure to high oxygen concentration of 9 hours compared to those managed expectantly. Spearman correlation revealed a significant positive correlation between delay to feeding and length of oxygen therapy (*r* = 0.87, *p* < 0.001, *N* = 87) but not with time to resolution or age at diagnosis.

**Table 3:** Comparison of neonates with pneumothorax and respiratory distress between expectant management and nitrogen washout therapy.

	Expectant management (*n* = 53)	Nitrogen washout (*n* = 34)	*p* value
Gestational age, w	39 [38–40] (34–42)	39 [37.8–39.3] (35–41)	0.146
Birth weight, g	3357.9 ± 514.1 (2060–4435)	3208.8 ± 479.5 (1990–4150)	0.179
Sex (female)	13 (24.5)	11 (32.4)	0.426
Primary event	44 (83)	31 (91.2)	0.352
Age at diagnosis, d	1 [1–1] (1–6)	1 [1–1] (1–3)	0.144
Length of oxygen therapy, h	0	9 [8–12] (6–85)	**<0.001**
Delay to first feeding, h	0 [0–0] (0–18)	13.5 [9–19.3] (0–25)	**<0.001**
Time to resolution, h	36 [23–64] (4–168)	33.5 [16–61.5] (8–189)	0.396
Length of hospital stay, d	4 [3–5] (2–22)	4 [3–6] (1–11)	0.375

Data are expressed by median [IQR] and (range), mean ± SD (range) or *n* (%).

Table 4 compares targeted oxygen therapy and nitrogen washout treatment in neonates diagnosed with pneumothorax who presented with desaturation in addition to respiratory distress. The rate of RDS was higher in the targeted oxygen therapy group compared to the nitrogen washout group (41.7% vs 7.7%, respectively, *p* = 0.009). There were no significant differences between treatments regarding GA, BW, gender ratio, MAS rate, age at diagnosis, length of hospital stays, length of oxygen therapy and delay to feeding. However, neonates treated with nitrogen washout had a significantly shorter median time to resolution compared to those treated with targeted oxygen therapy (31 vs 81 hours, *p* = 0.012). In stepwise linear regression, time to resolution was associated with length of oxygen therapy (*B* = 0.67, *t* = 4.64, 95%CI [0.4–1], *p* < 0.001) but not with RDS, MAS, BW, GA, gender, age at diagnosis and SP treatment. The model accounted for a moderate proportion of variance in time to resolution (R^2^ = 0.258, F(1, 63) = 21.52, *p* < 0.001).

**Table 4:** Comparison of neonates with pneumothorax, respiratory distress and desaturation between targeted oxygen therapy and nitrogen washout therapy.

	Targeted oxygen therapy (*n* = 12)	Nitrogen washout (*n* = 52)	*p* value
Gestational age, w	39 [35.3–40] (34–41)	39 [38–40] (36–42)	0.231
Birth weight, g	2976.8 ± 601.3 (1946–3925)	3291.5 ± 483.7 (1665–4445)	0.057
Sex (female)	6 (50)	15 (28.8)	0.185
RDS	5 (41.7)	4 (7.7)	**0.009**
Meconium aspiration syndrome	5 (41.7)	20 (38.5)	>0.99
Age at diagnosis, d	1 [1–1] (1–2)	1 [1–1] (1–10)	0.719
Primary event	2 (16.7)	28 (53.8)	**0.020**
Length of oxygen therapy, h	23.5 [8.5–54.8] (2–125)	11.5 [9.3–19.8] (6–240)	0.270
Delay to first feeding, h	24 [14–51.8] (10–72)	18 [11–23.5] (0–72)	0.109
Time to resolution, h	81[31.8–123.8] (18–138)	31 [12–67] (6–264)	**0.012**
Length of hospital stay, d	6 [4.5–15] (3–20)	5 [3–9] (1–31)	0.119

Data are expressed by median [IQR] and (range), mean ± SD (range) or *n* (%).

*RDS* Respiratory Distress Syndrome.

## Discussion

Our findings revealed that infants diagnosed with mild to moderate SP and presenting with respiratory distress had similar times to resolution and lengths of hospital stay when managed expectantly, without requiring oxygen treatment. Conversely, in infants with SP presenting with respiratory distress and desaturation, nitrogen washout treatment resulted in a significantly shorter time to resolution compared to targeted oxygen therapy, aligning with our hypothesis. This treatment showed similar time to first feeding, and lengths of oxygen therapy and hospital stay. This research stemmed from our routine challenge of treating pneumothorax in the NICU. Although nitrogen washout is widely used in many NICUs globally, evidence on its effectiveness in neonates remain limited. Additionally, prolonged exposure to high oxygen concentrations, which generates free radicals, increases the risk of adverse effects on various organs and tissues [23], especially in the neonatal population which is characterized by a suboptimal antioxidant defense system [14]. Susceptibility to oxidative stress correlates with neonatal development (SGA/AGA) and GA [24]. Prolonged exposure to high oxygen levels notably increases the risk of retinopathy of prematurity (ROP) in early preterm neonates and those with very low or extremely low BW, whereas this risk is much lower in late preterm and term neonates [24]. Balancing the toxic and beneficial effects of oxygen therapy in neonatal care emphasizes the need to define optimal oxygen levels and treatment approaches for SP, particularly in preterm infants. In this study, we aimed to determine the most appropriate approach to treating mild to moderate pneumothorax in late preterm and term neonates based on their clinical respiratory presentation. While most studies have primarily focused on the etiology of SP, few have discussed the use of oxygen therapy and its impact on SP resolution in neonates [20]. Clark et al. have shown that nitrogen washout had no superiority to targeted oxygen therapy in neonates above 35 weeks of gestation with small to moderate pneumothorax [25]. In another study, Shaireen et al. showed that there were no differences in the time to clinical resolution of SP among neonates treated either with room air or various concentrations of oxygen [20]. The clinical significance of our findings has prompted a reassessment of our local practices for managing mild to moderate SP in late preterm and term neonates. Our updated approach focuses on expectant management when oxygen saturation is adequate, while the non-invasive option of nitrogen washout is reserved for cases with desaturation, as it has been demonstrated to promote earlier resolution of SP. Furthermore, the observed delay in feeding initiation for neonates receiving oxygen therapy was largely due to the prevalent practice of postponing feeding during oxygen therapy, a practice that has recently been reassessed.

The gender effect seen in our cohort towards an overrepresentation of male neonates with SP has been previously described by others [26, 27]. Our cohort had a rather large proportion of cesarean deliveries. Similarly, Benterud et al. have shown an association between cesarean delivery and the incidence of SP [28].

The strength of our study lies in its relatively large cohort, spanning over a decade. Additionally, the comparison groups were mostly homogeneous in terms of maternal morbidities and medication, BW, GA, gender ratio, and age at diagnosis. However, some limitations should be noted, such as the retrospective nature of the study, the subjective description of respiratory symptoms, and the fact that it was conducted at a single center. Furthermore, the decision to provide oxygen therapy to neonates with adequate oxygen saturation may have been influenced by a more severe clinical presentation of dyspnea, compared to those managed expectantly, despite both groups having similar recovery times and hospital stays. In conclusion, nitrogen washout treatment was superior to targeted oxygen therapy in reducing mild to moderate SP time to resolution in infants presenting with respiratory distress and desaturation. On the other hand, when saturation was preserved nitrogen washout offered no advantages. Further studies are warranted to confirm our findings.

## Data Availability

The data that support the findings of this study are not openly available due to reasons of sensitivity and are available from the corresponding author upon reasonable request. Jackyh@tlvmc.gov.il.
